# No (true) right to die: barriers in access to physician-assisted death in case of psychiatric disease, advanced dementia or multiple geriatric syndromes in the Netherlands

**DOI:** 10.1007/s11019-023-10190-8

**Published:** 2024-02-20

**Authors:** Caroline van den Ende, Eva Constance Alida Asscher

**Affiliations:** 1 Department of General Practice/Family Medicine, Amsterdam UMC, Amsterdam, The Netherlands; 2 Department of Ethics, Law and Humanities, Amsterdam UMC, Amsterdam, The Netherlands

**Keywords:** Physician-assisted death, Euthanasia, Ethics, Right to die

## Abstract

Even in the Netherlands, where the practice of physician-assisted death (PAD) has been legalized for over 20 years, there is no such thing as a ‘right to die’. Especially patients with extraordinary requests, such as a wish for PAD based on psychiatric suffering, advanced dementia, or (a limited number of) multiple geriatric syndromes, encounter barriers in access to PAD. In this paper, we discuss whether these barriers can be justified in the context of the Dutch situation where PAD is legally permitted for those who suffer unbearably and hopelessly as a result of medical conditions. Furthermore, we explore whether there are options to address some of the barriers or their consequences, both within the Dutch legal framework or by adjusting the legal framework, and whether these options are feasible. We conclude that although there are insufficient arguments to overrule the doctor’s freedom of conscience in the Netherlands, there are ways to address some of the barriers, mainly by offering support to doctors that would be willing to support a request. Moreover, we believe it is morally required to reduce or mitigate where possible the negative consequences of the barriers for patients, such as the long waiting time for those who suffer from psychiatric disorders, because it is unlikely the adjustments suggested to the system will ensure reasonable access for these patient groups.

## Introduction

Although in many countries around the world the practice of physician-assisted death (PAD) remains under debate, the number of jurisdictions that have legalized or that are considering legalisation has increased over the last 20 years (Mroz et al. 2020). This leads to new questions and topics for discussion, such as the issue of ensuring reasonable access to all who are likely to be able to be helped while fulfilling the legal requirements for PAD (Shavelson et al. 2022). In this paper we discuss this issue from the context of the Netherlands, where PAD was legalized in 2002.

The Dutch legislation means that PAD is in principle a criminal offence, but that physicians are exempted from criminal liability if they comply with the due care criteria as defined in the Termination of Life on Request and Assisted Suicide (Review Procedures) Act (Euthanasia Act, see box 1) (De Haan 2002). The details of each completed case (of performed euthanasia) have to be reported to the Regional Euthanasia Review Committees, to ensure that the procedure has been carried out in accordance with these legal criteria.

**Table:** 

Box 1 Due care criteria from the Euthanasia Act (2002)
The physician must:
a. Be satisfied that the patient’s request is voluntary and well considered
b. Be satisfied that the patient’s suffering is unbearable, with no prospect of improvement
c. Have informed the patient about his situation and his prognosis
d. Have come to the conclusion, together with the patient, that there is no reasonable alternative in the patient’s situation
e. Have consulted at least one other, independent physician, who must see the patient and give a written opinion on whether the due care criteria set out in (a) to (d) have been fulfilled
f. Have exercised due medical care and attention in terminating the patient’s life or assisting in his suicide

Since the enactment of the Euthanasia Act there has been debate about the scope of the law. In the last decade this discussion focused especially on patients with a so-called complex request, such as a wish for PAD based on psychiatric suffering, advanced dementia, or (a limited number of) multiple geriatric syndromes. Among citizens there is strong support for the idea that everyone should have a ‘right’ to PAD. However, physicians' willingness to provide PAD to patients with complex conditions is less than that in the case of patients with a life-threatening physical condition such as cancer (ZonMW 2023). No physician can be compelled to fulfil a request for PAD and, as we will show in this paper, a ‘right to die’ does not exist in the Netherlands either. Reasons for this approach include the right of any physician to choose not to perform PAD (conscientious objection), but are broader than this: sometimes a physician is not convinced the due care criteria can be fulfilled, feels overburdened or is unwilling to perform PAD in particular cases. In 2012, the Dutch Voluntary Euthanasia Society founded the End-of-Life Clinic (as of 2019, ‘Euthanasia Expertise Center’, henceforth EEC) to give all patients with a request for PAD the opportunity to check whether the legal criteria can be fulfilled (and if this is the case/the criteria can be fulfilled, to receive PAD). This option is possible because there is no condition in the law (see above) of a prior treatment relationship between the physician and patient. The EEC, a network of physicians and nurse practitioners, acting as pairs, and employed by an institution based in The Hague, functions as a safety net in case of refusal of a request for PAD—especially for patients with complex requests (Expertisecentrum Euthanasie 2019a).

These more complex requests for PAD are ethically and/or legally more controversial (Bolt et al. 2015), which is reflected in the extra requirements case law has added (Verdict Supreme Court 1994, 2002, 2020a). In the case of requests based on psychiatric complaints and advanced dementia which raise concerns about the ability to make a voluntary and well-considered request, an additional independent physician with relevant disease specific knowledge must see the patient and establish competence as well as judge the possibility of alternative treatments to reduce the suffering (reasonable alternatives). For requests based on multiple geriatric syndromes it is necessary that the suffering is based on medically classifiable disorders in order to qualify for PAD.

Both the level of moral controversy and the additional requirements for complex cases may cause more hesitancy with the physician’s willingness to perform PAD, in contrast to requests based on suffering from a clear physical disease that makes death imminent (such as from terminal cancer patients), that are clearly granted more often (Regionale Toetsingscommissies Euthanasie 2022). This extra caution and as a result more difficult access to PAD for the complex cases seems, given their (moral) controversy, largely justified. However, the limited availability of doctors who are willing to assess or to perform PAD in these complex cases may result in de facto no access for these cases or extremely long waiting times.

In this paper, we will explore the barriers in access to PAD for the three aforementioned groups (psychiatry, advanced dementia, and multiple geriatric syndromes) and the consequences of these barriers. Where these barriers or consequences appear neither proportional nor reasonable we will discuss options to address some of them, both within the Dutch legal framework or by adjusting the legal framework. Finally, we will discuss whether these approaches are feasible and likely to improve access.

The question on how to ensure reasonably equal access to PAD is one that should be considered in other jurisdictions where PAD is considered acceptable. As such this case study of the Dutch experience and barriers may offer food for thought for other jurisdictions that have legalized PAD, but where some patients experience access issues.

## Psychiatric patients: various obstacles to access PAD

There are several areas in which physicians often have concerns when it comes to PAD for psychiatric patients, which makes it more challenging for these patients to be granted PAD. First, there are the concerns related to the (im)possibility to fulfil the due care criteria. In establishing that the request is voluntary and well-considered and not unduly influenced by the psychiatric disease, there are worries whether these patients can choose death voluntarily, whilst suicidality is often an aspect of their disease (ZonMW 2017). Furthermore, as the course and prognosis of psychiatric disorders are often less predictable, physicians differ with respect to the ability to assess the irremediability of suffering, and to establish that there are no reasonable treatment alternatives. For psychiatric disorders, the focus is not only on alternatives now, but also in the foreseeable future, perhaps related to the longer life expectancy (in comparison with, for example, patients with a palliative physical condition) (Evenblij et al. 2019). In other words, many psychiatrists find judging that this patient’s suffering is without prospect of relief harder. This causes the lifespan paradox, where the irremediability is questioned on the basis of the longer lifespan in which much development of drugs and treatments may occur as well as spontaneous remission, while the longer lifespan at the same time means that the unbearable suffering is likely to be present for a much longer time. It is possible to argue that the need for PAD—in a way—is more pressing when a lifetime of suffering awaits instead of a horizon of days to months.

Another argument concerning irremediability in the debate about PAD in psychiatric patients evolves around treatment refusal. A scoping review found that in several studies, a considerable proportion of psychiatric patients that requested or received PAD refused one or more treatment options. One might wonder whether suffering is irremediable as long as treatment options exist, but on the other hand whether it is reasonable (and effective) to ask patients to undergo treatments for which they often lack motivation (van Veen et al. 2020).

Besides the concerns about meeting the due care criteria and uncertainty about prognosis and treatment in psychiatric patients, doctor–patient interaction may be difficult in this group, especially in the case of personality disorders (Nicolini et al. 2020). Both the patient and the attending physician can unconsciously transfer feelings and attitudes, and this so-called transference and countertransference could influence decision-making. It is crucially important that the attending physician who is considering granting PAD is not joining the patient in a tunnel vision towards death. This makes the exploration of a request for psychiatric PAD more difficult.

In the Netherlands the number of psychiatrists willing to consider a request for PAD from their patients reduced between 1995 and 2016 (ZonMW 2017). As a result, many of the psychiatric patients who are granted PAD are helped by physicians from the EEC. For instance in 2022 from the 115 performed requests of psychiatric PAD, 65 were granted by EEC physicians (Regionale Toetsingscommissies Euthanasie 2023). The number of requests the EEC receives and the limited number of psychiatrists that work there, lead to long waiting lists (Expertisecentrum Euthanasie 2020).

Based on case law, the Regional Review Committees operationalize the necessary caution in case PAD arises from psychiatric suffering, by a consultation of an additional, independent psychiatrist to assess in particular the voluntariness of the request (competence), the irremediability of the suffering and (the absence of) possible reasonable alternatives for the relief of suffering. These additional safe guards for patients who predominantly suffer from psychiatric complaints are attempts to mitigate the valid concerns of physicians but present another obstacle to access to PAD for these patients (Regional Euthanasia Review Committees 2022; Verdict Supreme Court 1994). In addition to difficulties finding a psychiatrist willing to consider (and perform) PAD, there is a lack of psychiatrists able and willing to act as an independent consulted psychiatrist, which adds another barrier to physicians who are willing to engage with a PAD request from a psychiatric patient.

It seems to us reasonable that there are extra safeguards in place for PAD in psychiatric patients, including the extra expertise required with respect to irremediability and voluntariness. We also support the free choice of the individual physician whether or not to engage with and possibly grant PAD. However, we contend that the waiting times (for up to 2 years to be able to start exploring PAD with a psychiatrist from the EEC and additional waiting while an independent expert is found) are unjustifiable for patients who already consider their suffering unbearable and without prospect of relief. As a result we explore ways to limit the barriers with respect to the waiting time and/or reduce the suffering, while maintaining the higher levels of carefulness for this particular patient group.

## Possibilities to address barriers

Although many psychiatrists are unwilling to engage with requests from their patients, there are also psychiatrists that would be willing to support a request in an ideal case but are currently reluctant. To assist this group, they could be offered support and training from other psychiatrists who do perform PAD, and the EEC is trying to assist and coach these psychiatrists. It will take time though before this alternative leads to additional requests being addressed and it is unclear how many that will be and to what extent that will reduce the waiting times.

### Adjusting the legal framework

For patients whose PAD request is based on psychiatric suffering, the Dutch case law requires increased carefulness. Whilst there are good arguments for increased carefulness in this patient group, the question needs to be asked what barriers are proportional. For example, the way the increased carefulness is operationalised is through being seen by an independent (second opinion) psychiatrist who makes a judgement on the competence to request PAD and on the irremediability of the suffering. The outcome from this consultation can be that the request is or is not sufficiently voluntary or well considered. The focus is also strong on whether the second opinion psychiatrist concludes either that there are no more reasonable alternatives or that another therapy (often pharmacological) should be tried first. Subsequently, an independent physician in addition (as codified in the due care criteria) must be consulted on the fulfilment of the due care criteria. There is already some flexibility in combining both in one person. However, it could be considered instead to accept the second opinion psychiatrist as the independent consultation as required by law (providing the psychiatrist was able and willing to address all due care criteria in the consultation) in order to limit the time taken and the associated burden without doing concessions to the careful consideration of the request. This change might appear to reduce the barriers experienced by psychiatric patients, but the barrier is not really the consultation with the second independent physician: there is a well-established system to find an independent physician and there is usually very little additional waiting time. However, the most significant barrier is the limited number of psychiatrists willing to consider a request and this seems unlikely to change as a result of this adjustment. Adjusting the legal framework is unlikely to substantially reduce the waiting times and associated suffering for the patients.

### Mitigating some of the suffering while waiting

While there are more requests than can be dealt with by psychiatrists willing to explore a PAD request, the waiting lists for these psychiatrists will continue to exist. This waiting serves no purpose and prolongs suffering. If there are no effective ways to decrease the waiting time in the near future, it seems morally required to explore ways to make the waiting more bearable. Psychiatric patients describe that being taken seriously in their request for PAD and being able to discuss PAD openly preferably with their own physician is helpful (Pronk et al. 2022). Another approach might be through a palliative psychiatry approach or a project similar to the Reakiro House in Belgium (Reakiro 2020). Reakiro offers support to persons with a wish for PAD based on psychiatric suffering and their relatives. They can walk-in here for information or to discuss their wish. The assessment of a request for PAD falls outside the scope of Reakiro. Research is ongoing to investigate the effects of Reakiro on the suffering of the patients.

## Patients with advanced dementia: ethical concerns

The main ethical concerns with PAD for patients with dementia are related to competence and the possibility of making a voluntary and well-considered request. In the Netherlands, an earlier written advance euthanasia directive (AED) can replace an actual oral request for PAD. This means that people with advanced dementia who have lost the competence to ask for PAD or to confirm a previous request can still be granted euthanasia. However, in practice this option is very rarely realized. In for example 2022, six patients with advanced dementia were granted PAD on the basis of their AED, whilst 282 patients with dementia were granted euthanasia whilst still competent to request it (compared to a total of 8720 performed PADs in 2022) (Regionale Toetsingscommissies Euthanasie 2023). Where advance directives for treatment refusal are largely accepted, granting euthanasia based on an AED is much more controversial. In 2020 the Dutch Supreme Court confirmed that doctors in the Netherlands may grant PAD to patients with severe dementia based on an AED even when the patient can no longer express this wish. The decision followed a case in which a doctor was prosecuted after she carried out euthanasia on a patient with advanced dementia based on her AED, without oral confirmation of the request at the time of the euthanasia (Asscher & van de Vathorst 2020; Verdict Supreme Court 2020b). It was the first prosecution of a physician since the enactment of the Dutch Euthanasia Act in 2002, and the case received a lot of media attention. Although the doctor was finally acquitted, and the Supreme Court clarified the interpretation of the law, fear of prosecution still may prevent physicians from performing euthanasia in cases of late dementia.

Moreover, many doctors have personal objections related to PAD in patients with advanced dementia (Schuurmans et al. 2021). Even physicians who are quite willing to engage with challenging requests, may find performing PAD on an incompetent adult who at the time of the euthanasia cannot consent to the procedure and who has been unable to communicate about his/her death wish for a period of time, a moral line they are unwilling to cross (Schuurmans et al. 2019). Ending a life of an incompetent patient when they cannot verify the voluntariness of the request is problematic to them, and makes realizing euthanasia in these cases more burdensome. Another difficulty concerns the determination of unbearable suffering. Although suffering can manifest itself in non-verbal signals, doctors find it hard to conclude on unbearableness when verbal communication with the patient has become (almost) impossible (Kouwenhoven et al. 2015).

With advanced dementia there is an ethical debate on whether it is actually morally acceptable at all. However, it is hard to explain to patients (and their relatives) that the likelihood of being granted PAD is close to zero in the context of a country where this procedure is legally regulated. One could conclude that the legal provision creates a mostly theoretical option, considering the reluctance of the medical specialists concerned. The possibility to rely on an AED instead of an actual voluntary and well-considered request is the only part of the law that was not based on practice and case law. Alternatively one could argue that the formal possibility of PAD on the basis of an AED is misleading for patients with (early) dementia who might mistakenly postpone PAD till it is de facto too late. One conclusion might be this is a step too far, even in the Netherlands, as illustrated by a legal analysis of the Parliamentary introduction of the Euthanasia Law (Postma 2021), which would call for the withdrawal of this possibility. However, while the possibility is present, the difficulties experienced in access to PAD seem neither reasonable nor justified. Here we explore possible ways to lower the barriers experienced by patients with dementia, keeping in mind the necessary care for this ethically controversial patient group.

## Possibilities to address barriers

There are physicians willing to engage with PAD in incompetent patients, but who are unsure about the legal requirements and/or concerned about prosecution (de Nooijer et al. 2017). These doctors could be offered support by someone who is experienced with performing PAD in incompetent patients, as is a present policy measure of the EEC, where hesitant or uncertain physicians can find support by more experienced colleagues. The number of physicians willing to engage is low, however, and unlikely to increase substantially. Therefore it is important to inform patients who want to access PAD on the basis of dementia, that it is sensible to request PAD before the loss of their competence if they suffer unbearably as the likelihood of PAD being granted is much higher at that time.

### Adjusting the legal framework

In the case of PAD in patients with advanced dementia based on an AED, a doctor must consult at least two other doctors, including one with specialist knowledge of dementia as an additional requirement. Unlike with psychiatry there are no reports that this expertise is difficult to arrange.

Given the concerns about competence of these patients and the difficulty assessing unbearable suffering this greater caution is justified, although like with psychiatric patients questions may be asked about the proportionality of this extra caution. If the independent physician as required in the due care criteria has all the expertise necessary (is for instance a gerontologist with experience of this patient group), it is not unreasonable to conclude sufficient care has been taken without another second opinion. This adjustment is relatively small, but equally unlikely to make a large difference to the challenges in access. Moreover, considering the moral controversy with this patient group, it may not be prudent to change this.

If the fear of prosecution is the main barrier to performing PAD in this group, there may be possibilities to increase the certainty of the way a case is judged. Physicians often mention their wish for a review procedure before PAD in complex cases (ZonMW 2017). This option is not likely however as it would require a radical overhaul of the legal framework, which comes with downsides as well.

A final option would be to remove PAD from the realm of criminal law altogether. While this would address the fear of prosecution, and perhaps could in time be explored for the less complex cases of PAD, it is highly unlikely that it would be enacted for patients who no longer have the capacity to ask for PAD. In fact, there is legal discussion on the validity of AED based on international human rights law (Buijsen 2022; Cahill 2018; Rozemond 2020). Moreover, the physicians with moral and psychological objections are the majority and we believe they would not be encouraged by this change, nor welcome it. All in all there is very limited scope of changing the low likelihood of having euthanasia performed on the basis of an AED when competence is lost through dementia. This in any case at present needs to be completely clear to patients with early dementia and an AED.

## Patients with multiple geriatric syndromes: the grey area

Many physicians are hesitant with performing PAD in patients with multiple geriatric syndromes, and their main concern is that the request for PAD is based on being ‘tired of living’—which falls outside the scope of the Euthanasia Act (Bolt et al. 2015). With its ruling in the Brongersma case, the Supreme Court added a requirement to the second due care criterion related to the patient’s suffering: the suffering giving rise to the request for PAD must be *predominantly* the result of a classifiable medical condition (Verdict Supreme Court 2002). The Brongersma case concerned an 86-year old man with some age-related complaints such as osteoporosis and incontinence, but without a serious somatic illness or a psychiatric disorder. He suffered greatly though from loneliness, meaninglessness, increasing dependency and physical decline. His physician was satisfied that his suffering was unbearable without prospect of improvement—and granted his request for PAD. According to a Lower Court the patient’s suffering was mainly caused by a lack of life perspective and the Supreme Court ruled that this did not justify PAD. Although a physician who is considering granting PAD may also assess psychosocial or existential factors that play a role in the request, legally these factors need to be the result of a medical complaint caused by a medically classified affliction and cannot be the major stand-alone cause of suffering. In practice, it can be difficult for doctors to determine whether the cause of suffering is predominantly medical in older persons, as patients tend to put more emphasis on the existential factors (van den Berg et al. 2021). Furthermore, the distinction between ‘normal’ decline in the process of ageing and medical disease seems rather arbitrary (Rurup et al. 2005), and the concept of unbearable suffering is personal. What is acceptable and bearable for one patient, may not be acceptable and unbearable for another, and—especially with old age afflictions—doctors may not always understand the patient’s suffering.

Whereas one of the implications of the Brongersma case appears to be that being ‘tired of life’ is insufficient as a basis for physicians to carry out PAD, and most doctors have difficulties with PAD in this group, in society there is greater support for making PAD possible for elderly people who are tired of life, but not seriously ill. In 2016 one of the parties in the Dutch parliament announced its intention to create a member’s bill that regulates PAD for this group. The proposed law on ‘end of life guidance for the elderly’ (Tired of living law) includes lawful assisted suicide by so called end-of-life-counsellors (not necessarily being physicians) for all those over 75 with a well-considered request. The PERSPECTIEF study, conducted to gain more knowledge on the group of elderly with a death wish without being seriously ill, showed that such a group exists but is not limited to the over 75s (about 10,000 persons of 55 years and older). Furthermore, although the respondents do not consider themselves seriously ill, the vast majority suffers from many physical and mental complaints (Wijngaarden et al. 2020). One could question how many of those could in theory receive PAD on the basis of the existing law (because they have sufficiently severe suffering based predominantly on a medical condition). However, this is not straightforward even for patients that could be granted euthanasia while fulfilling the due care criteria.

The proportion of PAD requests granted by a doctor from the EEC that involved patients with multiple geriatric syndromes has increased between 2013 and 2019 (Expertisecentrum Euthanasie 2019b; Levenseindekliniek 2014, 2015, 2016, 2017, 2018). Perhaps attending physicians refer those patients to the EEC, because they believe the request cannot be granted as they cannot fulfil the due care criteria, assuming the patients are predominantly tired of life (van der Meer 2018). Between 2013 and 2018 about 48–70% of requests for PAD based on multiple geriatric syndromes were actually granted by a doctor of the EEC (Expertisecentrum Euthanasie 2019b; Levenseindekliniek 2014, 2015, 2016, 2017, 2018). Presumably the other requests could not be granted on the basis of being unable to fulfil the due care criteria, such as the unbearable suffering criterion because of insufficient causal relation with a medically qualified disease. In other cases patients may have decided against PAD or died before PAD could be performed. However, it does indicate that access to PAD is difficult for this group. If PAD is allowed for these patients, any barriers should be reasonable and related to relevant differences between these patients and others that can access PAD more easily. Currently the barriers however do not seem justified by relevant ethical differences, as they seem to be due to uncertainty of the scope of the law.

## Possibilities to address barriers

The main reason physicians are more hesitant with requests from PAD from patients with multiple geriatric syndromes is, as we discussed above, related to the requirement from case law that the suffering should be predominantly based on a medically classified affliction. Physicians want to avoid requests based on being tired of life where there are insufficient medical afflictions, but what sufficient and predominantly mean in this context is unclear. Whilst education and coaching by those who are experienced with this group might encourage PAD in those fulfilling the criteria, the fact is there are shades of grey in this group and many physicians would rather avoid those, if criminal prosecution is a threat.

### Adjusting the legal framework

The easiest solution for the grey area would be to remove the requirement that the unbearable suffering is based on a medically classifiable problem. Morally there is little difference between the suffering from one or another source. However, although physicians complain about the grey area, they would likely not welcome this change, as they are not uniquely qualified to deal with non-medical suffering, and they are currently the only group allowed to perform PAD. Moreover, it is fair to ask whether it would be reasonable to ask physicians to deal with requests outside the medical realm as well.

Another option for this group would be, if the proposed Tired of living law would be enacted. The Council of State has looked into this proposal and concluded it doesn’t contain sufficient safeguards as it stands and so advices against enactment (Raad van State 2022). An adjusted law might not fall short and allow all those over 75 (or another age criterion) to end their life with assistance.

## Discussion and conclusion

In this paper we showed that also in the Netherlands, where PAD is allowed (under conditions) for many years now, a ‘right to die’ with assistance does not exist. Doctors have the right to conscientiously object to perform PAD if this conflicts with their personal and moral beliefs, or may refuse a request for PAD for other reasons, for example when they are not convinced that the due care criteria can be met. In most cases there is another physician available after referral who may consider the request: the Netherlands is a small country after all and the EEC fills this vacuum in part with the availability on a national scale of physicians with more experienced assessment of the issue on fulfilment of the due care criteria. However, even the availability of this selected group of physicians does not guarantee reasonably equal access. Certain groups, especially the complex cases such as those with psychiatric disorders, advanced dementia and multiple old-age complaints, encounter real barriers when attempting to access PAD. These differences in access—in comparison with other groups such as those with requests based on suffering from a clear physical disease—are to a certain extent justified, considering the higher level of moral controversy and complexity in (the exploration of) these requests for PAD. However, we observe that these cases currently have hardly any access to PAD. Although we believe that there are no convincing arguments to overrule the concept of conscientious objection—granting PAD is burdensome to doctors and they should be able to weigh their own considerations (Savulescu 2006; Schuklenk 2015)—this difference in access seems unjust, given the context of a jurisdiction where PAD is permitted for these groups. This does not mean that every patient should have the right to die with assistance, but it seems justified to lower where possible the barriers and/or reduce the differences in access. In all of the three discussed patient groups offering support and education to doctors who are willing to perform PAD in these cases (but are for example unsure about the legal requirements) could encourage those physicians to consider PAD for patients with more complex requests that can meet the legal criteria. Also, we are morally required to relieve the (unnecessary) suffering that is caused by the long waiting times for in particular psychiatric patients, and explore ways that can make this more bearable for these patients. Adjusting the legal framework, for example by changing the current review procedure to a procedure before PAD in complex cases (to assist those physicians that are predominantly deterred by possible legal consequences), is (legally) very complex. Also the more rigorous option of removing euthanasia from the domain of criminal law, remains extremely controversial and is unlikely to happen.

In conclusion, although none of the options we explored will completely remove the barriers and result in sufficient access (as many doctors still have moral concerns related to PAD), there are some possibilities, mainly within the legal framework, to ensure more reasonable access to PAD for patients with psychiatric disorders, dementia and multiple old-age complaints when in principle PAD is permitted. Where the access is likely to remain problematic, necessary approaches include good palliative psychiatric care, and clear information about the low chance of euthanasia on the basis of an AED for patients hoping to die that way. One could even consider alternative access to assistance in self-chosen death for the elderly in order to limit the suffering.
